# Laminar circuit organization and response modulation in mouse visual cortex

**DOI:** 10.3389/fncir.2012.00070

**Published:** 2012-10-05

**Authors:** Nicholas D. Olivas, Victor Quintanar-Zilinskas, Zoran Nenadic, Xiangmin Xu

**Affiliations:** ^1^Department of Anatomy and Neurobiology, School of Medicine, University of CaliforniaIrvine, CA, USA; ^2^Department of Biomedical Engineering, University of CaliforniaIrvine, CA, USA; ^3^Center for Complex Biological Systems, University of CaliforniaIrvine, CA, USA; ^4^Department of Electrical Engineering and Computer Science, University of CaliforniaIrvine, CA, USA

**Keywords:** GABA, NMDA receptor, voltage-sensitive dye imaging, optical stimulation, PCA

## Abstract

The mouse has become an increasingly important animal model for visual system studies, but few studies have investigated local functional circuit organization of mouse visual cortex. Here we used our newly developed mapping technique combining laser scanning photostimulation (LSPS) with fast voltage-sensitive dye (VSD) imaging to examine the spatial organization and temporal dynamics of laminar circuit responses in living slice preparations of mouse primary visual cortex (V1). During experiments, LSPS using caged glutamate provided spatially restricted neuronal activation in a specific cortical layer, and evoked responses from the stimulated layer to its functionally connected regions were detected by VSD imaging. In this study, we first provided a detailed analysis of spatiotemporal activation patterns at specific V1 laminar locations and measured local circuit connectivity. Then we examined the role of cortical inhibition in the propagation of evoked cortical responses by comparing circuit activity patterns in control and in the presence of GABAa receptor antagonists. We found that GABAergic inhibition was critical in restricting layer-specific excitatory activity spread and maintaining topographical projections. In addition, we investigated how AMPA and NMDA receptors influenced cortical responses and found that blocking AMPA receptors abolished interlaminar functional projections, and the NMDA receptor activity was important in controlling visual cortical circuit excitability and modulating activity propagation. The NMDA receptor antagonist reduced neuronal population activity in time-dependent and laminar-specific manners. Finally, we used the quantitative information derived from the mapping experiments and presented computational modeling analysis of V1 circuit organization. Taken together, the present study has provided important new information about mouse V1 circuit organization and response modulation.

## Introduction

The primary visual cortex (V1) is considered as the first cortical stage for visual information processing and has been extensively studied as the classic cortical model system (Nassi and Callaway, 2009; Gao et al., 2010). More recent efforts are devoted toward understanding functional connections and response dynamics in local and micro-cortical circuits of V1 (Dantzker and Callaway, 2000; Ohki et al., 2005; Yoshimura et al., 2005; Bock et al., 2011). As the mouse is genetically amenable and more readily accessible than large mammals (e.g., primates and cats) and because the basic organization of the mouse visual cortex is remarkably similar to that of primates (Gao et al., 2010; Wang et al., 2012), the mouse has become an increasingly important animal model for the studies of visual cortical circuits (Niell and Stryker, 2008, 2010; Runyan et al., 2010; Andermann et al., 2011; Marshel et al., 2011). However, even compared to rat visual cortex (Burkhalter, 1989; Shao and Burkhalter, 1996; Yuste et al., 1997; Dantzker and Callaway, 2000; Yoshimura et al., 2005; Zarrinpar and Callaway, 2006) as well as mouse auditory, somatosensory, and motor cortex (Shepherd et al., 2003, 2005; Oviedo et al., 2010; Hooks et al., 2011), there are few studies that have examined anatomical and functional laminar circuit connections in mouse visual cortex (Wang and Burkhalter, 2007; Xu et al., 2010; Wang et al., 2011). Therefore, in the present study, we have investigated mouse V1 laminar circuit responses and their functional modulation by mainly using our newly developed mapping technique which incorporates laser-scanning photostimulation with voltage-sensitive dye (VSD) imaging (Xu et al., 2010; Xu, 2011).

Photostimulation-based mapping techniques have been widely applied for analyzing cortical circuits. Laser scanning photostimulation (LSPS) combined with whole cell recording is an effective method for mapping local circuit inputs to single recorded neurons (Callaway and Katz, 1993; Schubert et al., 2003; Shepherd and Svoboda, 2005; Weiler et al., 2008; Xu and Callaway, 2009). LSPS has also been combined with two-photon calcium imaging to generate detailed functional maps of inputs to individual cells with single-cell and three-dimensional precision (Nikolenko et al., 2007). Different from the aforementioned approaches, the mapping technique used in the present study is intended to assess circuit activation and network connectivity at the neuronal population level through fast VSD imaging and photostimulation (Xu et al., 2010; Xu, 2011). During experiments, LSPS using caged glutamate offers spatially restricted neuronal activation in a specific cortical layer such that functional projections from the stimulated layer to its targeted layer(s) are detected by VSD imaging of evoked activation. The new approach allows for a network-level assessment of neuronal population responses across V1 laminar circuits.

Specifically, the present study first investigate V1 circuit connections and spatiotemporal dynamics of circuit responses in living brain slices, which was built on and expanded from our previous preliminary examination (Xu et al., 2010). Furthermore, as GABAergic inhibition affects the response properties of visual cortical neurons and modulates spatiotemporal spread of population activity (Tanifuji et al., 1994; Nelson and Katz, 1995; Yuste et al., 1997; Ferster and Miller, 2000; Katzner et al., 2011), we examine and compare laminar specific patterns of cortical responses in normal control ACSF and bath application of GABAa receptor antagonists. We would like to determine how GABA receptors shape excitatory signal propagation in V1 laminar circuits and address the role of GABAergic inhibition in restricting layer-specific excitatory activity spread. In addition, as it is important to examine how ionotropic glutamate receptors (NMDA and AMPA receptors) differentially contribute to visual cortical circuit connections and signal propagation, we investigate the effects of specific glutamate receptor antagonists on evoked neuronal population responses. It has been reported that these receptors differentially contribute to generation of neuronal spikes (Armstrong-James et al., 1993) and functional magnetic resonance imaging signals (Gsell et al., 2006) in sensory cortex *in vivo*. Moreover, glutamate receptors are differentially distributed in the various layers of the neocortex (Aoki et al., 1994; Dodt et al., 1998). *In vitro* VSD imaging also shows that there is differential distribution of NMDA and AMPA receptor activity in layer II/III in mouse V1 slices in response to layer IV electrical stimulation, as the AMPA receptor signal was strongest in the middle of layer II/III and the NMDA receptor signal was strongest at the layer I/layer II border (Bellinger and Hensch, 2005). Therefore, we also use our mapping approach to determine whether there is layer-specific modulation of circuit responses via these receptors. Finally, based on the quantitative information derived from our mapping experiments, we present computational modeling analysis of V1 circuit responses.

## Results

### Mouse V1 laminar circuit responses and interlaminar excitatory signal propagation

**Figure 1:**
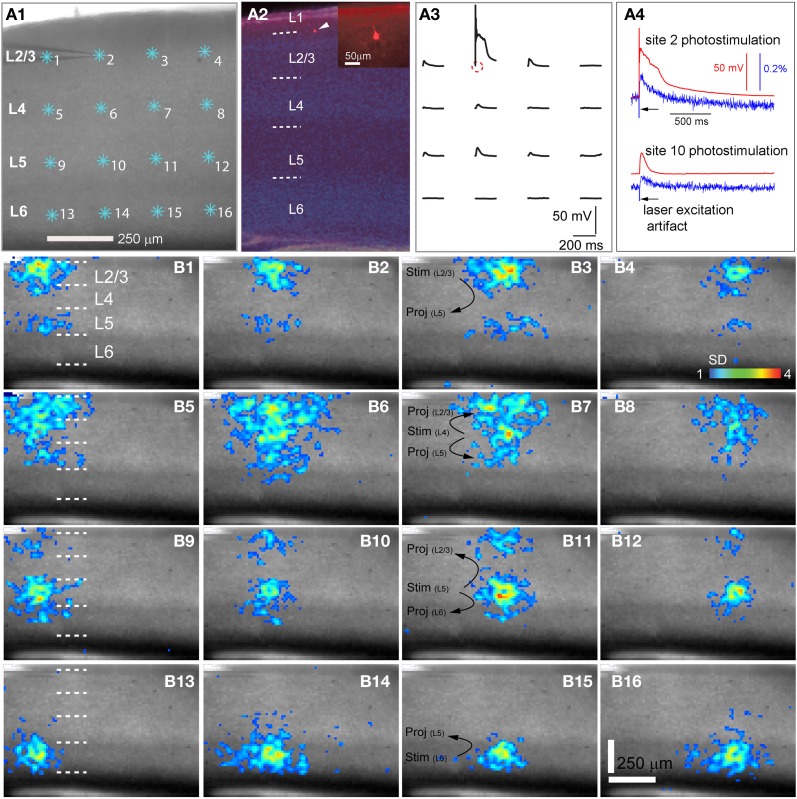
**High precision and fast mapping of local V1 laminar circuit organization through laser scanning photostimulation and voltage-sensitive dye (VSD) imaging. (A1)** Shows the slice image with cyan asterisks indicating a 4 × 4 stimulus pattern covering V1 from cortical layer 2/3 to layer 6. The glass microelectrode was recording from a single pyramidal neuron in the upper layer 2/3 during photostimulation and imaging for monitoring the spatial precision of laser photostimulation and correlating single-cell activity with the VSD signal of the population response. **(A2)** Shows the DAPI-stained image of the same V1 slice recorded in **A1**, with an overlay image of the cell stained against biocytin (pointed by the white arrowhead). The insert in **A2** shows the morphology of the recorded pyramidal cell under a higher magnification. The short dashed white lines in **A2**,**B1,B5,B9** and **B13** denote the laminar boundaries of cortical layers 1, 2/3, 4, 5, and 6. **(A3)** Indicates spatially restricted neuronal activation via photostimulation by plotting the changes in the membrane potential of the recorded neuron in response to photostimulation of the 4 × 4 sites shown in **A1**. The small circle indicates the cell body location. **(A4)** Shows data traces of simultaneous whole-cell recording and VSD imaging in response to photostimulation at sites of 2 and 10, respectively. Red traces represent membrane potentials of the recorded neuron, and blue traces represent VSD signals that were measured from a small region (4 × 4 pixels) around the electrode tip shown in **A1**. The black arrows in **A4** point to the artifact of laser excitation in the VSD signal traces. **(B1**–**B16)** Show peak activation maps from the VSD image sequences, at each of the 16 stimulation sites indicated in **A1**. The map of peak activation is derived from a single image frame with peak VSD response. VSD signal amplitudes expressed as standard deviations (SD) above the mean baseline signal are color coded. The map pixels with amplitudes ≥1 SD are plotted over the slice image. Warmer colors indicate greater excitation. The main projection patterns for the photostimulated cortical layers are plotted at **B3**,**B7**,**B11**, and **B15**. Note that the CCD camera images have a slightly different aspect ratio. Under the 4× objective, the camera covers an area of 1.28 (*w*) × 1.07 (*h*) μm^2^ with a spatial resolution of 14.6 (*w*) × 17.9 (*h*) μm^2^/pixel. The same conventions are used in the following figures.

**Figure 2:**
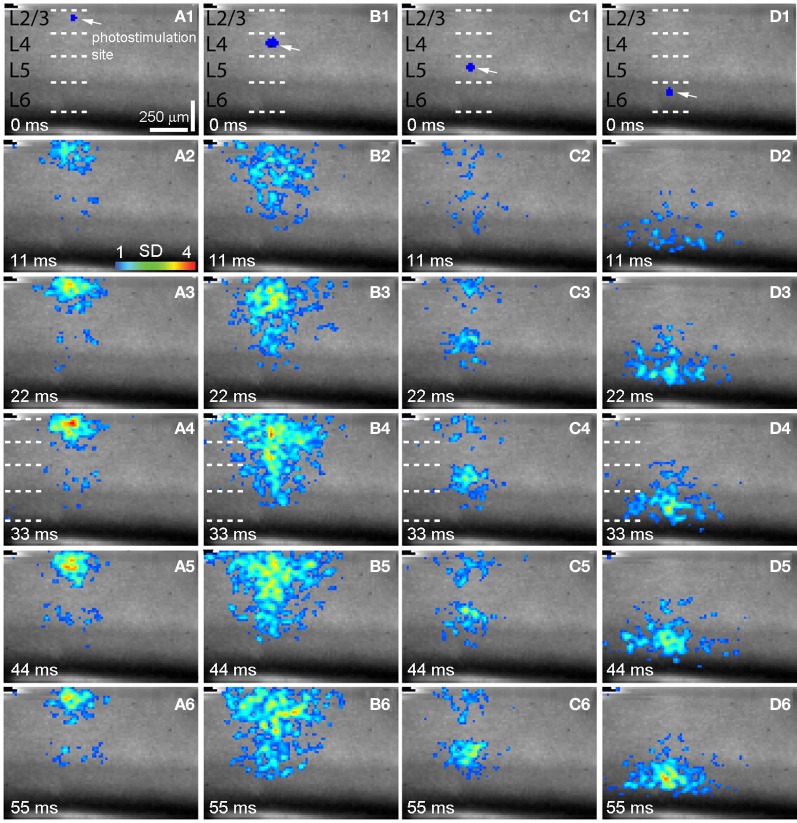
**Spatiotemporal distribution of photostimulation-evoked VSD responses across V1 laminar circuits. (A–D)** are time series data of imaging photostimulation-evoked responses at V1 cortical layers 2/3, 4, 5, and 6 (i.e., sites 2, 6 10, 14 of the same slice as shown in Figure 1A), respectively. VSD images were acquired at the rate of 2.2 ms/frame during the experiment and are displayed at the specified time points. Time progresses from top to bottom in the column, and color code is used to indicate VSD signal amplitudes expressed as standard deviations (SD) above the mean baseline. The site of photostimulation can be identified by the laser excitation artifact (indicated by the white arrow) in the initial frame of the sequence. The short dashed white lines in **A1–D1** denote the laminar boundaries of V1 layers 2/3, 4, 5, and 6.

**Figure A1:**
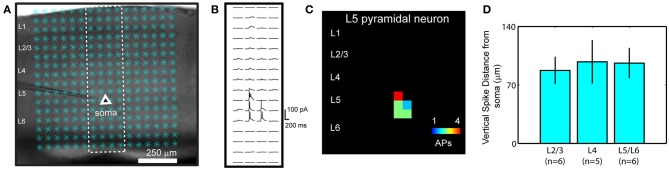
**Spatial precision of laser scanning photostimulation (LSPS). (A)** Illustration from mapping a photostimulation-evoked excitation profile by recording a layer 5 neuron in the current clamp mode while photostimulation is delivered on the mapping grid (16 × 16 sites, each spaced at 65 μm^2^). The cell soma is indicated as a triangle. **(B)** Membrane potential traces in response to photostimulation at the white dashed box in A (4 × 16). Suprathreshold responses were evoked at the proximal and perisomatic region of the recorded neuron, whereas subthreshold responses resulted from distal dendritic activation. **(C)** The number of action potentials per uncaging location surveyed by LSPS from the representative pyramidal neuron. **(D)** LSPS evoked spikes are within the home layer of the recorded neurons. The data plotted (mean ± SE) are the average vertical distances from the somata, where action potentials were evoked for layer 2/3, 4, and 5/6 cells, respectively.

The functional mapping technique of combined LSPS and fast VSD imaging, as detailed previously (Xu et al., 2010; Xu, 2011), was used to examine V1 local circuit responses. We have chosen to use VSD imaging over Ca^2+^ imaging, because of the concerns including the lack of robust signal detection through low-power objectives and the pitfall that not all neuronal types with action potentials produce measurable Ca^2+^ transients (Knopfel et al., 2006). In our experiments, photostimulation and imaging were performed through a 4× objective, with a field of view spanning the whole V1 coronal slice. In normal conditions (unless specified otherwise), laser photostimulation (20 mW, 1–2 ms) offered spatially restricted neuronal activation, and only neurons located close to photostimulation sites fired action potentials (Figures A1 A–C). The average vertical distances of photostimulation-evoked spikes from the recorded cell bodies were 87.4 ± 16.3 (mean ± SE) μm, 97.7 ± 25.9 μm, and 96.2 ± 18.2 μm, respectively, for layers 2/3, 4, and 5/6 cells (*N* = 17 total) (Figure A1 D). These data indicate that LSPS evoked spikes are within the home layer, thus stimulation precision allowed us to map direct projections from the photostimulated layer to its targeted layer(s) by VSD imaging of evoked activation. Photostimulation-evoked action potentials propagated through the axons of the stimulated neurons and generated postsynaptic subthreshold responses in the neurons that were connected to the stimulated cells (Figures 1, 2). The measured VSD signals reflected the combined contributions of these sources, but responses distant from the photostimulation site were dominated by postsynaptic changes rather than activity in the axons and distant dendrites of directly stimulated cells. This was evidenced by control experiments in which VSD signals were restricted to the region near the stimulation site when synaptic transmission was blocked by using low Ca^2+^ and high Mg^2+^ ACSF or when synaptic spread and conduction of activity with the axons of stimulated cells were blocked by TTX (Xu et al., 2010).

As our previous paper was published as an innovative methodology article (Xu et al., 2010) and has not fully described spatiotemporal response profiles or the features of laminar circuit connectivity, here we expand upon our initial description of mouse V1 circuit mapping. VSD responses evoked by photostimulation across different laminar locations were spatially discrete with laminar specific topography. Figure 1 shows the overall laminar profile of photostimulation-evoked VSD responses in V1 by presenting peak activation maps at each of the 16 stimulation sites distributed from cortical layer 2/3 to layer 6; Figure 2 displays individual VSD image sequences in response to laser photostimulation at representative sites of cortical layers 2/3, 4, 5, and 6. The average width of the response profiles at the peak activation for layers 2/3, 4, 5, and 6 was 138.7 ± 13.2 μm, 284.7 ± 60.6 μm, 116.8 ± 13.8 μm, and 160.6 ± 23.1 μm, respectively (*N* = 5–8 slices). The general patterns of VSD responses have been described in the earlier work (Xu et al., 2010) and detailed more below. While strong VSD responses to photostimulation occurred in layer 2/3, the primary output of layer 2/3 stimulation was targeted to layer 5, bypassing most of layer 4 with weak excitation in layer 6 (Figures 1B and 2A, Table 1A). Photostimulation in layer 4 produced most strong and robust excitatory activity that spread vertically to layers 2/3 and 5, but without much excitation descending to layer 6 (Figures 1B and 2B, Table 1A). Layer 5 photostimulation yielded a reciprocal ascending projection pattern in contrast to the descending projection of layer 2/3 to layer 5 (Figures 1B and 2C, Table 1A). Layer 5 activation was also spread into layers 6 and 4. Compared to layers 2/3, 4, and 5, photostimulation in layer 6 only resulted in weak postsynaptic responses in the more superficial layers of V1; and most response propagation was detected in layer 5 (Figures 1B and 2D, Table 1A). We further examined the temporal aspect of interlaminar propagation of VSD responses. The temporal onset of the VSD responses amongst layers 2/3, 4, 5, and 6 were 14.7 ± 1.5 ms, 16.7 ± 1.4 ms, 28 ± 1.4 ms, and 29.7 ± 1.1 ms, respectively (*N* = 8–12 for each laminar location). The propagation time for which layer 2/3 VSD activation robustly spread into layer 5 was 28 ± 1.9 ms (*N* = 8). The propagation time for layer 4 VSD responses getting to layers 2/3 and 5 was 24 ± 0.74 ms and 31 ± 1.27 ms, respectively. In addition, the propagation time for layer 5 to layer 2/3 was 37.7 ± 1.2 ms.

**Table 1:** **VSD activation in response to laminar specific photostimulation, and interlaminar functional projection strength in normal ACSF**.

**(A) LAYER-SPECIFIC VSD ACTIVATION IN PHOTOSTIMULATED AND OUTPUT LAYERS**^**a**^					
**Photostimulated cortical layer**	**Laminar specific activated pixels**				**Total activated pixels across layers 2/3–6**
	**Layer 2/3**	**Layer 4**	**Layer 5**	**Layer 6**	
Layer 2/3	61.17 ± 10.92 (mean ± SE)	20.32 ± 6.77	68.67 ± 23.91	17.77 ± 6.7	167.92 ± 26.58
Layer 4	187.91 ± 44.7	175.59 ± 31.76	150.73 ± 30.14	53.14 ± 9.98	569.01 ± 93.85
Layer 5	35.05 ± 15.51	19.31 ± 5.38	90.44 ± 12.45	38.79 ± 8.01	221.76 ± 64.75
Layer 6	2.14 ± 0.42	2.18 ± 0.4	10.25 ± 4.08	83.4 ± 14.95	97.57 ± 17.87
**(B) QUANTITATIVE DATA OF INTERLAMINAR FUNCTIONAL PROJECTION STRENGTH**^**b**^					
**Photostimulated cortical layer**	**Interlaminar projection strength (Q**_**proj**_)			
	**Layer 2/3**	**Layer 4**		**Layer 5**	**Layer 6**
Layer 2/3		0.29 ± 0.10 (mean ± SE)		0.48 ± 0.16	0.19 ± 0.50
Layer 4	1.38 ± 0.33			0.86 ± 0.13	0.31 ± 0.18
Layer 5	0.76 ± 0.3	0.45 ± 0.15			0.47 ± 0.11
Layer 6	0.02 ± 0.0	0.05 ± 0.01		0.12 ± 0.02	

a Photostimulation-evoked VSD activation was quantified by the average number of activated pixels in the image frame at the defined peak response phase. Data quantification for each laminar location was based upon 7–11 VSD image datasets.

b See the section “Materials and Methods” for quantification of interlaminar functional projection strength (Q_proj_).

Given the quantitative measurement of evoked cortical activation and the estimated number of action potentials generated by photostimulation in specified V1 layers, this study allowed for quantification of functional interlaminar projection strength (Q_proj_) (see the “Materials and Methods” for details). Briefly, Q_proj_ accounts for the number of postsynaptically activated neurons relative to the number of action potentials generated by presynaptic neurons per photostimulation.

$$Q_{\text{proj}}=N_{\text{postsynaptically activated neurons}}/N_{\text{presynaptic AP}}.$$

The total number of activated neurons (*N*_postsynaptically activated neurons_) was determined by the total activated pixel volumes and the average neuronal density across mouse V1 (Schuz and Palm, 1989). The number of action potentials generated by presynaptically activated neurons via LSPS (*N*_presynaptic AP_) was quantified by (ρ_cell_ × V_exc_ × S_AP_) (Shepherd et al., 2005), where ρ_cell_ denotes the neuronal density at the location of glutamate uncaging, V_exc_ represented the volume of excited tissue by laser pulse, and S_AP_ is the number of action potentials fired by cell per laser pulse. As summarized in Table 1B, the average projection strength Q_proj_ of layer 2/3 → layer 5 was determined to be 0.48 ± 0.16, which indicated the presynatic spiking by layer 2/3 neurons (~267 spikes per photostimulation) would activated about 128 layer 5 neurons in general. The average Q_proj_ of layer 2/3 → layer 5 was larger than that of layer 2/3 → layer 4 or layer 6 (*P* < 0.05). The Q_proj_ of layer 4 → layer 2/3 was 1.38 ± 0.33, the largest among all interlaminar projections (*P* < 0.05), while that of layer 4 → layer 5 or 6 was 0.86 ± 0.13 and 0.31 ± 0.18, respectively. The size and amplitude of VSD response spread from layer 4 photostimulation to layer 2/3 was consistent with the Q_proj_ of layer 4 → layer 2/3. Compared to layer 2/3 → layer 5 projection, the Q_proj_ of layer 5 → layer 2/3 appeared larger (*P* < 0.05), being 0.76 ± 0.3. The Q_proj_ of layer 5 → layer 4 or 6 was 0.45 ± 0.15 and 0.47 ± 0.11, respectively. Given that layer 6 did not have strong output as noted above, the Q_proj_ was quite weak across all layers.

**Figure 3:**
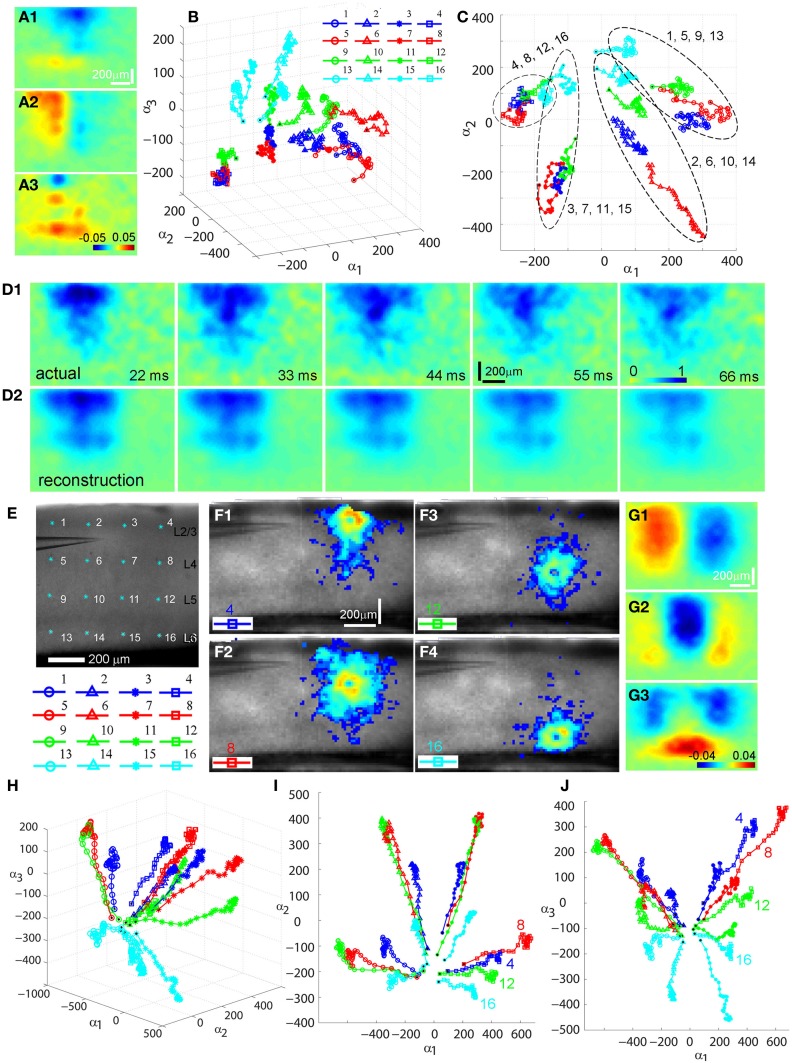
**Principal component analysis (PCA) of photostimulation-evoked VSD responses. (A–D)** and **(E–J)** present PCA of two different VSD image data sets. **(A–D)** are from PCA of the data set as shown in Figure 1. **(A1–A3)** Shows the first three principal spatial modes (shown as images) derived from the PCA decomposition of the combined VSD image sequence from the 16 stimulation sites (Figures 1 B1–B16) (See the section “Materials and Methods”). The first 30 time frames (66 ms) of the image sequence after photostimulation were used for analysis. **(B)** Shows the time varying expansion coefficients (trajectories) of each of the three principal modes in a 3D phase space. Each curve corresponds to one of the 16 responses from different stimulation sites, with the marker color indicating the row position of the site related to V1 cortical layers (layer 2/3-blue, layer 4-red, layer 5-green, layer 6-cyan), and the marker shape indicating the column position of the photostimulation site (from left to right: circles, triangles, stars and squares corresponding to the 1st, 2nd, 3rd and 4th column sites). The black dot indicates the trajectory start. See the insert for the indicated map sites. **(C)** Shows the 2D projection of the trajectories, shown in the plane α_1_ − α_2_. Four trajectory groups can be identified with each of these groups gathered by their column locations of photostimulation (i.e., sites 1, 5, 9, 13; sites 2, 6, 10, 14; sites 3, 7, 11, 15; sites 4, 8, 12, 16). **(D1)** and **(D2)** illustrate the validity and effectiveness of PCA for the VSD imaging data. **(D1)** Shows the image sequence generated from the original VSD response (site 6 in Figure 1). Note that the map images represent difference images with a pre-photostimulation background frame subtracted, and darker colors code stronger VSD response. **(D2)** Shows reconstructed image sequence based on the five principal modes of the global basis (obtained by combining datasets from all the 16 VSD responses from different map sites). With this PCA basis, the five principal modes were sufficient to capture 75.37% of the total variance for all 16 stimulation responses; the breakdown of the variance captured by the five principal modes is 30.51%, 24.87%, 8.92%, 6.71%, and 4.35%, respectively. The first three principal modes are plotted as images in **(A1–A3)**. **(E–J)** are from PCA of similar V1 mapping experiments as in Figure 1 except with strong laser photostimulation (3 ms, 35 mW) at each site. **(E)** Shows a portion of V1 slice image with asterisks indicating a 4 × 4 stimulus pattern spanning V1 layer 2/3 to layer 6. The insert below denotes the 16 map locations corresponding the 4 × 4 pattern with different colors and markers. **(F1–F4)** Are peak activation maps in response to photostimulation at the specified map sites (i.e., 4, 8, 12 and 16), respectively. **(G1–G3)** Show the first three principal spatial modes of the combined VSD image sequence. The five principal modes were sufficient to capture 80.02% of the total variance for all 16 stimulation responses; and the breakdown of the variance captured by the five principal modes is 39.25%, 15%, 12.55%, 8.08%, and 5.32%, respectively. **(H)** Shows a 3D phase space plot of the time varying trajectories. **(I)** Shows the 2D projection of the trajectories, shown in the plane α_1_ − α_2_. Four trajectory groups are clearly identifiable by their map column locations. Note that the trajectories from the same column at sites 4, 8, 12, and 16 are clustered at the lower right of the plot. **(J)** Shows the 2D projection of the trajectories, shown in the plane α_1_ − α_3_. It appears that the projection trajectories are arranged to preserve the laminar topography of stimulation sites (cyan, layer 6-bottom; green, layer 5-middle; red, layer 4 and blue, layer 2/3 -top). Note that the trajectories of sites 4, 8, 12, and 16 from the same column but at different cortical layers are separately arranged from top to bottom at the plot.

We further analyzed photostimulation-evoked VSD responses in V1 circuits by applying principal component analysis (PCA) to VSD image data. The decomposition method such as PCA has been effectively used for analyzing neuroimaging data (Senseman and Robbins, 1999, 2002; Nenadic et al., 2002, 2003). Detailed analysis of high-dimensional VSD imaging data in the original data domain can be difficult and even impractical. However, the analysis process can be substantially simplified with PCA, as the global features of a coherent signal such as waves of propagating neural activity are typically captured by low-dimensional spatial modes while the majority of noise remains in the higher order modes which are discarded by PCA. For the present study, PCA was effective in allowing quantitative comparisons of two or more photostimulation-evoked VSD data sets, which would boil down to comparisons of their low-dimensional phase space trajectories since the spatial modes are common to all data sets (Figure 3). The validity and effectiveness of PCA for the photostimulation evoked VSD responses is illustrated in Figures 3 D1,D2, which plot actual VSD response images and reconstructed images with a small number of principal components. For this particular example, Figure 3 D1 plots the image frames of the actual VSD response to photostimulation in V1 layer 4, and Figure 3 D2 plots its PCA reconstruction. While the global basis was constructed from a very diverse set of data (combination of responses from the 16 different map sites), the five principal modes (particularly the first three) were sufficient to capture essential information for the reconstruction of individual responses. The total captured variance by the principal modes and reconstruction quality depended on the extent and strength of interlaminar propagation of photostimulation evoked responses. This is expected, as PCA better extracts stronger propagation activity that results in a more coherent wave dynamics. Figure 3 presents PCA of two V1 cases with different photostimulation strengths. Compared to Figures 3A–D, the V1 case with stronger response propagation is shown in Figures 3E–J.

Several interesting features of spatiotemporal dynamics emerged from the PCA examination across experimental data sets. While the spatial modes of VSD response represent mathematical constructs (i.e., eigenvectors of the data covariance matrix), they exhibit columnar and laminar structures (Figures 3 A1–A3, G1–G3). These spatial modes may underlie vertical projections and upper and lower laminar profiles of photostimulation-evoked VSD responses. We also found that temporal trajectories of VSD response were related to their vertical projection columns and laminar locations of photostimulation across the cases examined (*N* = 4). Each trajectory in Figures 3 B,H describes the direction and speed of the VSD signal propagation of each of the 16 stimulation responses. These three-dimensional trajectories are separated and grouped with certain patterns, which are more clear when two-dimensional trajectory projections are considered. For the first case shown in Figure 3C, in the α_1_–α_2_ plane, four trajectory groups can be distinguishable and clustered by their vertical columns of photostimulation locations. This organization pattern is better defined in the second case with stronger response propagation. Note that the temporal trajectories of VSD responses originating in cortical layers 2/3, 4, 5, and 6 within the same vertical column (shown in Figures 3 F1–F4) are arranged into a distinct group at the lower right of Figure 3I. Furthermore, for the second case, in the α_1_ − α_3_ plane, the projection trajectories are arranged so that the laminar topography of stimulation sites tends to be preserved (Figure 3J). In addition, the separation of trajectories of the left two columns and the right two columns of the stimulation sites is visible as the symmetry in the α_1_–α_2_ and α_1_–α_3_ planes. The visualization of these features can further be aided by the multi-dimensional scaling plot which shows that the trajectories originating from the sites of the same column have a strong tendency to be clustered together (data not shown). Therefore, PCA further indicated that the spatiotemporal propagation of evoked VSD responses in V1 circuits was determined by photostimulation locations and aligned by vertical columnar connections.

### GABAergic modulation of VSD response propagation

**Figure 4:**
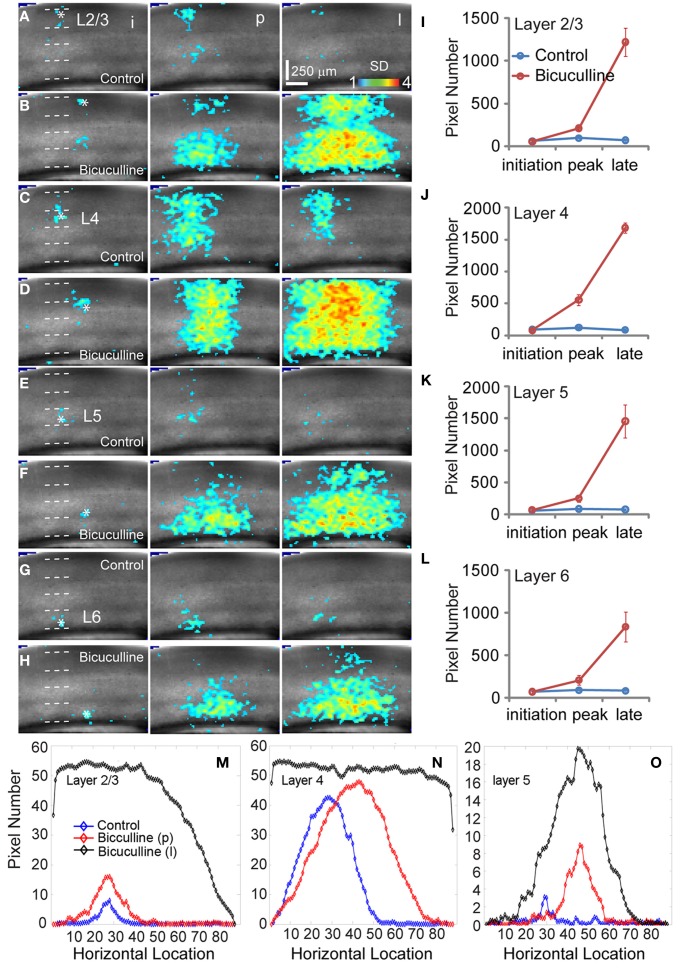
**Effects of antagonizing GABAa receptor activity with bicuculline on the spatiotemporal dynamics of local V1 circuit activity. (A**,**C**,**E,** and **G)** show individual image frames corresponding with the initiation (i), peak (p), and late phase (l) of the VSD activation, in response to photostimulation in normal ACSF in layers 2/3, 4, and 5 of a V1 slice, respectively. In comparison, **(B**,**D**,**F**, and **H)** show activation frames of the same slice re-mapped with the addition of bicuculline in ACSF, evaluated at the three temporal phases of the VSD activation, respectively. Note that 1 ms photostimulation was used for control, while 0.5 ms photostimulation was used in the bicuculline presence. **(I**–**L)** Show the average number of activated pixels at the initiation, peak, and late phases of control and bicuculline treated slices (*N* = 2). Data are presented as means ± SE. When the datasets of five slices were pooled, the response differences between control and blocking cortical inhibition were significant statistically (*P* < 0.05) at the peak and late phases across all the layers, but not significant for the initial phase (*P* > 0.5). **(M–O)** Show slice activation profiles of the peak phase of control, and the peak and late phases of bicuculline application for layers 2/3, 4, 5, and 6, respectively. For the plots, the *x* axis indicates horizontal location of activated pixels from the left to right of the image frame (in the unit of pixel); the y axis denotes the average number of column-wise summed activated pixels across image frames at the defined phases.

**Figure 5:**
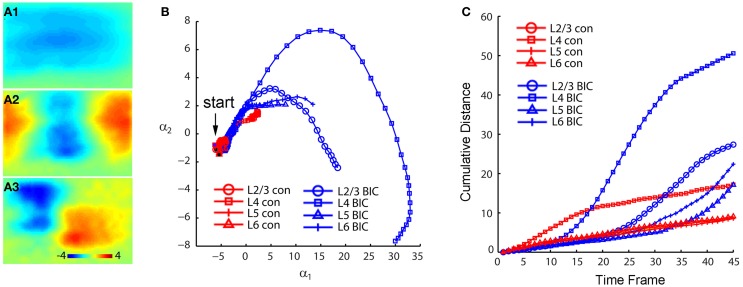
**PCA indicates that photostimulation-evoked VSD responses have much stronger propagation spread across V1 circuits with GABAergic inhibition blocked.** The PCA are based on the image data sets presented in Figure 4. **(A1–A3)** Shows the first three principal spatial modes (shown as images) derived from the PCA decomposition of the combined VSD image sequence (*N* = 8 files) from the four stimulation sites (layers 2/3, 4, 5, and 6) in control and in the presence of bicuculline. The first 45 time frames (99 ms) of the image sequence after photostimulation were used for analysis. **(B)** Shows the 2D projection of the trajectories of VSD responses, shown in the plane α_1_ − α_2_. **(C)** Shows the cumulative propagation distance of the time varying trajectories of VSD responses in the 3D phase space (α_1_, α_2_, α_3_).

When we mapped V1 circuit responses in the slices under normal conditions with intact inhibition, interlaminar excitatory signal propagation reflects the combined effect of local circuit excitation and inhibition. In order to reveal the effects of cortical inhibition on circuit activity, we did further experiments by comparing circuit activity patterns in control and with GABAa receptor antagonists (bicuculline or gabazine) to block GABAergic inhibition. We found that GABAergic inhibition was crucial in controlling spatiotemporal propagation of photostimulation-evoked VSD responses. GABAa receptor antagonists did not clearly affect early phase (11–33 ms after photostimulation) of VSD responses across laminar location, as the initial spatiotemporal patterns of the VSD responses were similar between control and pharmacological conditions (Figure 4). However, photostimulation-evoked VSD responses were stronger, lasted longer, and propagated over much larger cortical regions with the presence of GABAa receptor antagonists (Figure 4). The increase in spatial spread and prolonged duration of the VSD responses were observed in all layers. The supragranular and infragranular regions, which in control did produce very strong propagation of excitation, were strongly activated in the presence of GABAa receptor antagonists even by weaker photostimulation. In control conditions, VSD responses across all laminar locations reached peak levels at the defined peak phase (55–77 ms after photostimulation), and then decayed at the defined late phase (99–121 ms after photostimulation), remaining vertical interlaminar projections. In the presence of GABAa receptor antagonists, however, the VSD responses continued to increase and propagate both laterally and vertically over much larger regions even at the late phase (Figure 4). Based upon the response size and amplitude, blocking inhibition should have recruited new excitatory connections into the activated circuit, rather than simply revealing the direct extent of photostimulation-evoked excitatory connections. Under this condition, the average spread size of VSD responses between the peak and late phases for layers 2/3, 4, 5, and 6 was 506 ± 202.5 μm, 1095 ± 80.4 μm, 299.3 ± 167.9 μm, and 204.4 ± 73 μm, respectively (*N* = 6–8 per laminar site). With cortical inhibition blocked, the vertical propagation of VSD responses was clearly followed by a horizontal spread of excitatory activity across all V1 layers; VSD propagation from all sties exhibited no clear borders or discontinuity. This fits with the PCA of VSD responses, indicating stronger projection trajectories with longer propagation distance with GABAa receptor antagonists (Figure 5). On average, the propagation distance of response trajectories (in the defined phase space) in the presence of bicuculline or gabazine was 222.7 ± 17.4% relative to control (*P* < 0.05; *N* = 5). Thus, with loss of intracortical inhibition, excitatory activity spread failed to be constrained by V1 circuit topography as maintained in the control condition.

### Differential contributions of NMDA and AMPA receptors to neuronal population responses

**Figure 6:**
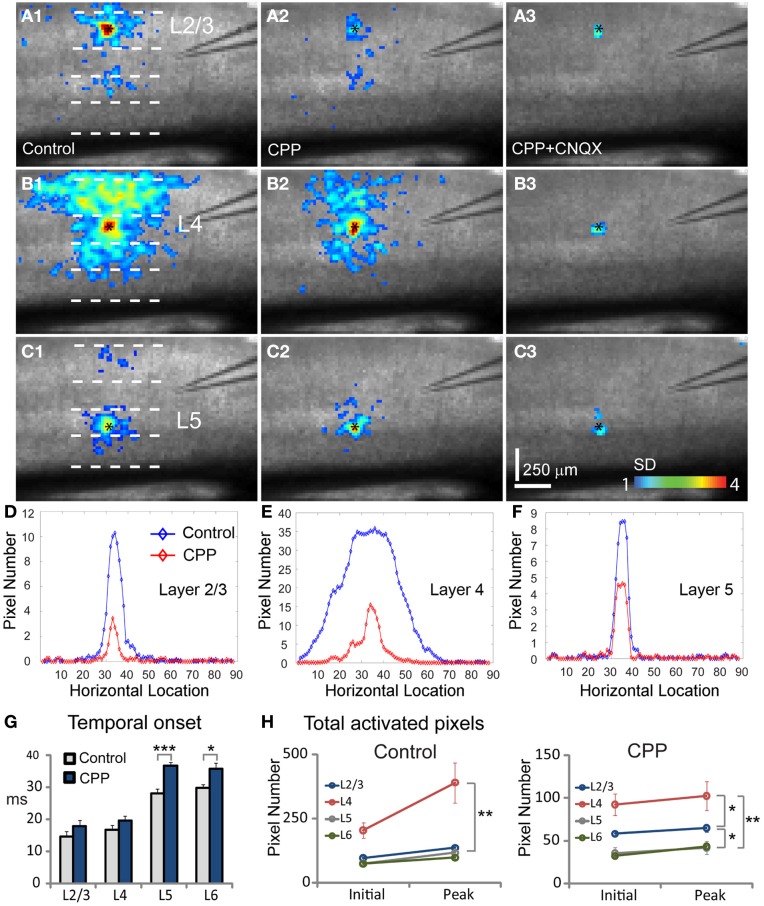
**Effects of blocking NMDA and AMPA receptor activity on neuronal population responses in V1 circuits.** Neuronal population responses evoked by photostimulation were detected by fast VSD imaging in normal ACSF, bath application of the NMDA receptor antagonist CPP, and co-application of CPP and the AMPA receptor antagonist CNQX. **(A1**,**B1**, and **C1)** Are peak activation maps of VSD responses in normal ACSF to photostimulation in layers 2/3, 4, and 5, respectively. **(A2**,**B2**, and **C2)** Are the peak activation maps in the presence of CPP to photostimulation in layers 2/3, 4, and 5 of the same slice, respectively. **(A3**,**B3**, and **C3)** are the peak activation maps in the presence of CPP and CNQX. Photostimulation sites are indicated by small black asterisks. **(D**,**E**, and **F)** Show activation profiles of peak VSD responses under control and CPP conditions for layers 2/3 **(A1,A2)**, 4 **(B1,B2)**, and 5 **(C1,C2)**, respectively. For the plots, the *x* axis indicates horizontal location of activated pixels from the left to right of the image frame (in the unit of pixel); the *y* axis denotes the average number of column-wise summed activated pixels across image frames at the peak response phase. **(G)** Plots temporal onsets of VSD responses in control and CPP conditions. **(H)** Shows the size of VSD responses quantified by the average number of activated pixels across 10 frames in the initiation phase and across 10 frames in the peak phase, for both control and CPP treated slices. Data are presented as mean ± SE. ^*^P < 0.05. ^**^P < 0.01. ^***^P < 0.001.

To address how the two types of ionotropic glutamate receptors differentially contributed to V1 functional circuit connections and excitatory activity propagation, we examined and compared neuronal population responses evoked by laser photostimulation or microelectrode electric stimulation in normal ACSF, bath application of the NMDA receptor antagonist CPP or the AMPA receptor antagonist CNQX, and co-application of CPP and CNQX (Figure 6). We found that AMPA receptor activity played a dominant role in mediating excitatory information flow in V1 circuits; NMDA receptor activity exerted an important modulation on functional connections and spatiotemporal dynamics of local population activity with response time-dependent and laminar specific manners.

During experiments, the effects of NMDA and AMPA receptor activation were isolated by pharmacologically blocking different types of receptors. For the photostimulation experiments, application of the NMDA receptor antagonist, CPP delayed the initiation of excitatory activity, as the temporal onset of VSD responses for layers 2/3, 4, 5, and 6 were 17.8 ± 1.8 ms, 19.6 ± 1.4 ms, 36.6 ± 1.0 ms, 35.8 ± 1.9 ms (*N* = 7–8 slices), increasing their response latency by about 21%, 18%, 31% and 20% with respect to control, respectively (Figure 6G). The CPP application also increase interlaminar propagation times, as the propagation time for layer 2/3 → layer 5, layer 4 → layer 2/3 and layer 4 → layer 5, and layer 5 → layer 2/3 was 36.8 ± 1.5 ms, 27 ± 0.7 ms, 34.1 ± 1.3 ms, 40.2 ± 2.5, respectively. Overall, there was 10–20% increase of interlaminar propagation time across V1 locations. Because NMDA receptor antagonists interfere with glutamate uncaging (which analogously mimics the release of glutamate naturally, although at a higher concentration), the longer onsets and increased propagation delays could be due to the fact that reducing NMDA receptor activity by CPP may cause some neurons at both the stimulation and projection sites to be activated below the detection threshold. Compared to normal ACSF conditions (Figures 6 A1–C1), the CPP application clearly reduced interlaminar and intralaminar propagation of photostimulation-evoked responses from all laminar locations (Figures 6 A2–C2). This was evidenced by the quantification shown in Figure 6H. The size of response profiles were significantly reduced (Figures 6D–F), and the average width for layers 2/3, 4, 5, and 6 was 92.5 ± 4.9 μm, 111.9 ± 12.8 μm, 73 ± 5.3 μm, and 94.9 ± 4.2 μm, respectively (*N* = 6 per laminar site). To exclude a major interference effect of CPP with laser photostimulation, we also performed VSD imaging of electric stimulation-evoked neural activity in cortical layer 4, and found similar CPP effects seen in the photostimulation experiments. As for electrical stimulation trials in the presence of CPP, the average peak response strength was 72.7 ± 5.3%, 76.5 ± 6.8%, 63.9 ± 4.9% for layers 2/3, 4, and 5, respectively, relative to control (*N* = 4 slices).

**Figure 7:**
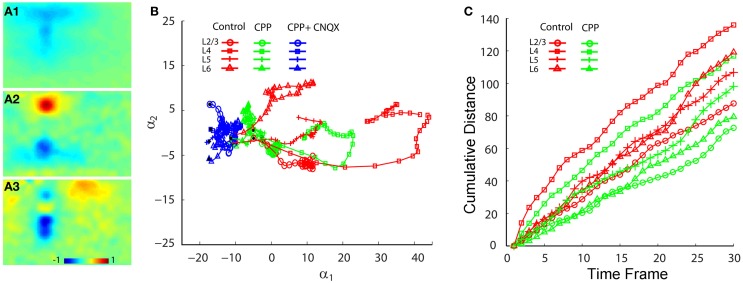
**PCA comparison of projection trajectories of photostimulation-evoked VSD responses in normal ACSF, in the presence of CPP and with the co-application of CPP and CNQX.** The PCA are based on the image data sets presented in Figure 6. **(A1–A3)** Show the first three principal spatial modes (shown as images) derived from the PCA decomposition of the combined VSD image sequence (*N* = 12 files) from the four stimulation sites (layers 2/3, 4, 5, and 6) in control (normal ACSF), in the presence of CPP and with both CPP and CNQX. The first 30 time frames (66 ms) of the image sequence after photostimulation were used for analysis. **(B)** Shows the 2D projection of the trajectories of VSD responses, shown in the plane α_1_ − α_2_. **(C)** Shows the cumulative propagation distance of the time varying trajectories of VSD responses (in control and in the presence of CPP) in the 3D phase space (α_1_, α_2_, α_3_).

Co-application of the AMPA receptor antagonist, CNQX, with CPP completely abolished all interlaminar functional projections, leaving only small residual responses in photostimulation sites (Figures 6 A3–C3). To determine whether AMPA receptors mediated the interlaminar excitatory transmission, we reversed the order of antagonist application in a separate series of experiments. Indeed, application of CNQX alone completely abolished translaminar propagation and blocked functional interlaminar projections across V1 laminar locations in both photostimulation and electric stimulation experiments (*N* = 6 slices). The VSD responses were only localized at the site of photostimulation, with the total activated pixels accounting for about 5–10% of the size of those pixels activated in the control. The general effects of application of CPP, and co-application with CNQX can also be seen in the PCA of VSD responses (Figure 7), as CPP reduced and the co-application of CPP and CNQX suppressed the propagation of their trajectories in the defined phase space.

**Figure 8:**
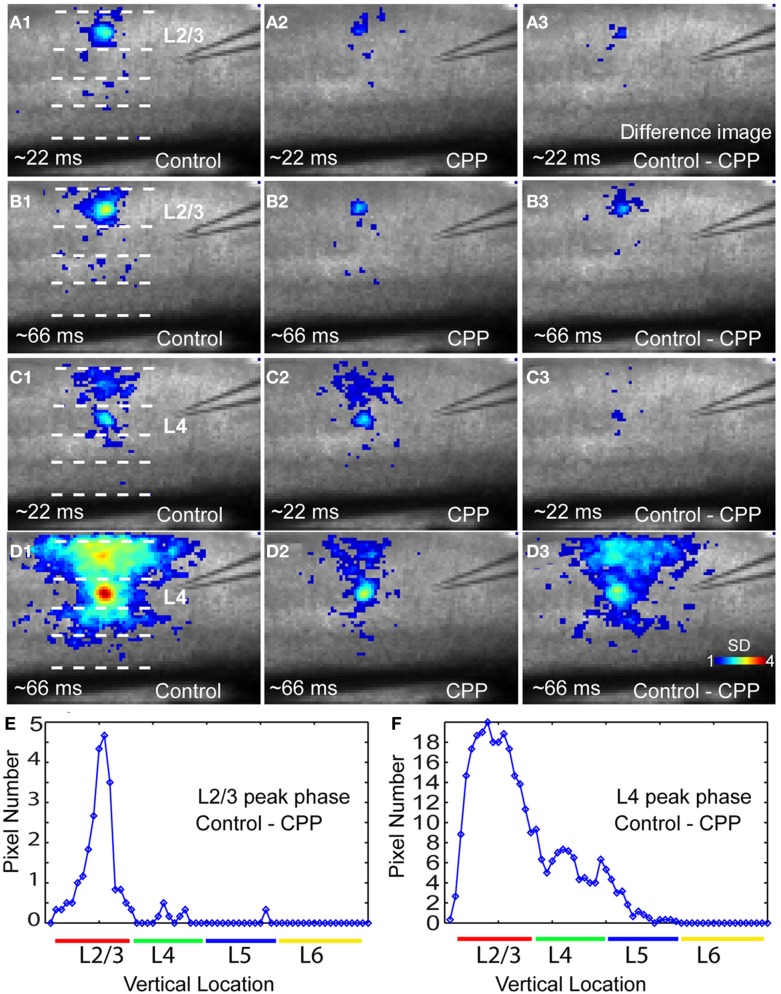
**The NMDA receptor antagonist CPP preferentially reduces peak activation of photostimulation-evoked VSD responses in a laminar-specific manner. (A1** and **A2)** are the averaged images across the initiation phase of VSD responses to photostimulation in layer 2/3 in normal ACSF and CPP, respectively. **(A3)** is the difference image between **(A1** and **A2)**. In these images, only the pixels with amplitudes ≥1 SD are plotted. **(B1** and **B2)** are the averaged images across the peak phase of VSD responses to photostimulation in layer 2/3 in normal ACSF and CPP, respectively. **(B3)** is the difference image between **(B1** and **B2)**. **(C1** and **C2)** Are the averaged images across the initiation phase of VSD responses to photostimulation in layer 4 in normal ACSF and CPP, respectively. **(C3)** Is the difference image between **(C1** and **C2)**. **(D1** and **D2)** are the averaged images across the peak phase of VSD responses to photostimulation in layer 4 in normal ACSF and CPP, respectively. **(D3)** is the difference image between **(D1** and **D2)**. **(E** and **F)** Show laminar distributions of image pixels in **(B3** and **D3)** at the peak phase, respectively. The *x* axis indicates the vertical location of image pixels from layer 2/3 to layer 6 (from the top to bottom of the image); the *y* axis denotes the number of row-wise summed pixels of the image.

In addition, closer examinations indicated that blocking NMDA receptors via the CPP application modulated neuronal population responses in time-dependent and layer-specific manners. As illustrated in Figure 8, CPP preferentially suppressed excitatory propagation around the peak response phase instead of the initial phase. The earlier initial phase was not so much affected by the presence of CPP while the large reduction took place at the peak phase. This reduction was apparent in the difference images of VSD activation between control and CPP (Figures 8 B3,D3). Moreover, the CPP effect on VSD responses at the peak phase was most pronounced in upper cortical layers, as layer 2/3 had more extensive reduction of VSD responses in terms of activation areas and amplitudes compared to deeper layers (Figures 8 B3,D3,E, and F). On average, in the CPP presence, layers 2/3 and 4 accounted for 44.3% ± 2.2% and 32.4% ± 1.4% of the total reduction of VSD responses to layer 4 photostimulation, while 22.7% ± 1% of the reduction occurred in layers 5, respectively (*N* = 4–10 per laminar location).

### Computational modeling analysis of V1 circuit responses

**Figure 9:**
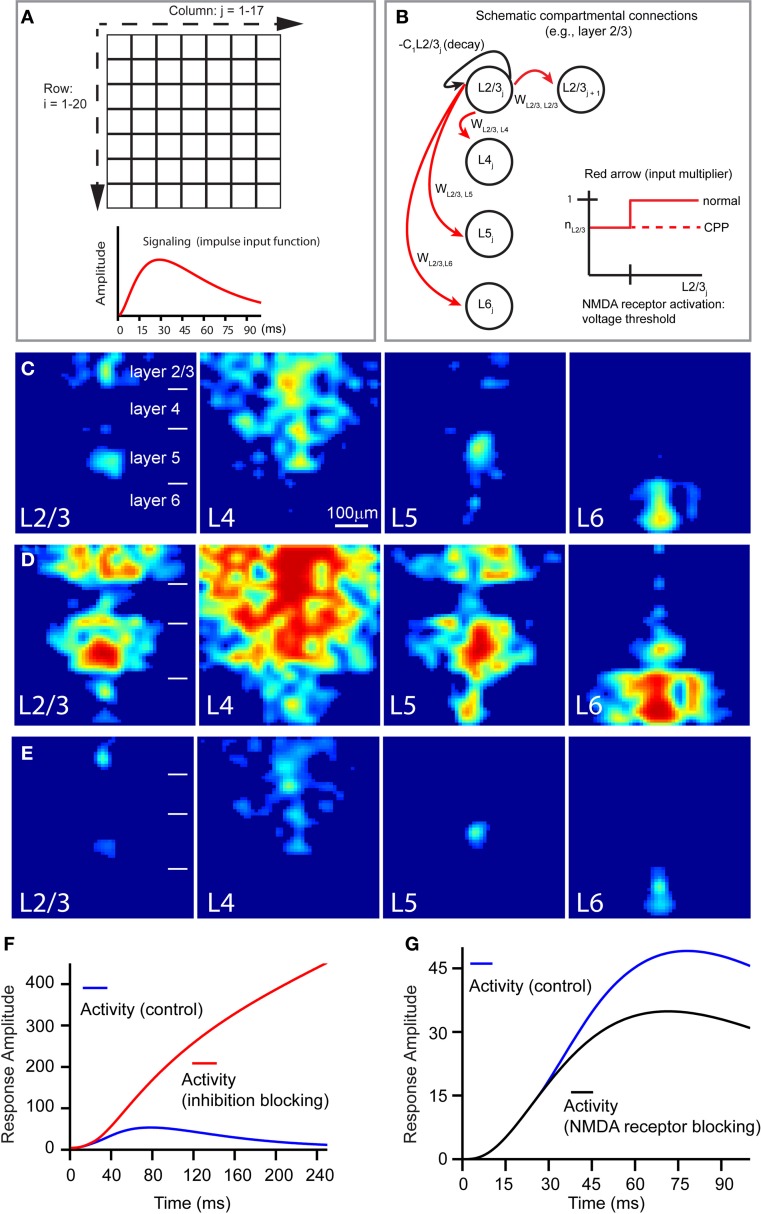
**Computational model of a V1 slice's response to laser photostimulation. (A,B)** Illustrate the general structure of our model slice. The model in **(A)** consists of a 20 × 17 grid compartment, with each compartment putatively corresponding to a 40 μm × 40 μm region of a V1 slice. The thicknesses of modeled cortical layers reflect their biological sizes relative to each other; compartment rows 1–4, 5–8, 9–14, and 15–20 correspond to layers 2/3, 4, 5, and 6, respectively. The 20 rows span about the whole cortical depth from layer 2/3 to layer 6, while the 17 columns (680 μm) are wide enough to simulate column-wise responses. Laser stimulation, modeled as a decaying exponential input approximating photostimulation, is “aimed” at a specified compartment, which in turn broadcasts to other compartments; the impulse input depicted below the grids. In the schematic shown in **(B)**, the following features of the model are illustrated: compartmental connections with layer-specific connection weights, compartmental activity decay, and NMDA-mediated voltage-dependence of compartmental responsiveness to input. **(C)** Shows the simulated slice's peak activation spread (~70 ms) in response to stimulation of L23, L4, L5, and L6, respectively. **(D)** Shows the late stage (~150 ms) of slice activation profiles with inhibition blocked. **(E)** Is same as **(C)**, except in the absence of NMDA conductance. **(F** and **G)** Show how the temporal evolution of overall system activity produced by layer 4 stimulation is affected by loss of cortical inhibition and NMDA receptor blocking, respectively, relative to control.

To extend our experimental results, we built a compartmental computational model of a V1 slice based on the derived quantitative information with the following features. First, spiking—and therefore, signal broadcast—happens only at the site of laser stimulation, and excitatory signaling scales nonlinearly with the compartment's activation. Second, the connection strengths between compartments reflect layer identity and are informed by our quantification of interlaminar projection strength (Table 1B). To better mimic random noise observed in experimental dye images, a noise term was added to these connection strengths. Third, compartmental activity negatively feeds back onto itself, putatively representing a combination of self-inhibition and spontaneous activity decay (due to repolarization), with blocking cortical inhibition (i.e., the application of bicuculline or gabazine) implemented as a reduction in negative feedback's overall strength. Fourth, to implement NMDA receptor activity, a fraction of a compartment's excitatory input is voltage-sensitive in the control condition and absent during simulation with NMDA receptor blocking (i.e., the CPP application). The compartmental model concept and some of the above features are illustrated in Figures 9A,B; model details are described in the section “Materials and Methods.”

Overall, this neural model successfully reproduced the activity spread observed under the control condition (Figure 9C), largely capturing the geometric and quantitative features of the observed activity spreads in the presence of GABAa receptor antagonists (Figures 9D,F), and in the presence of NMDA receptor antagonists (Figures 9E,G). When V1 responses were simulated in the absence of cortical inhibition, overall system activity was dramatically increased, without dropping off in the late phase (Figure 9F), resembling the experimental data (Figure 4). The model also reflected the mild effect of CPP on the early phase of the slice stimulation response, corroborating our image data and supporting the notion that this is due to the NMDA receptor activity being gated by voltage-sensitive thresholds. As seen in Figure 9G, the trajectories of overall system activity, with and without NMDA receptor activation, are indeed similar in the early time period (<30 ms), but in the later time period (40–65 ms), voltage-dependent activation of NMDA current is sufficient to cause overall system activity to continue rising higher than in the condition without NMDA receptor activation.

## Discussion

### Technical considerations

Our technique of combining fast dye imaging and LSPS, enabled effective mapping of spatiotemporal patterns and functional connections of local V1 circuits. Compared to earlier VSD imaging studies of V1 circuits with electrical stimulation or puffed glutamate (Tanifuji et al., 1994; Yuste et al., 1997), our all-optical approach for stimulation and imaging greatly improved the ability of assessment of evoked circuit activity. LSPS allowed for quantitative laminar and columnar mapping analysis (Shepherd et al., 2003; Shepherd and Svoboda, 2005; Xu and Callaway, 2009). It also permitted rapid evaluation of multiple locations, and avoided the interpretation difficulties associated with electrical stimulation of neural tissue. In addition, well-controlled photostimulation and quantitative imaging measurements allowed for quantification of projection strength between functionally connected regions. Although the imaging method permits us to map network activity at neuronal population levels, it does not have a single cell resolution and cannot allow us to map circuit inhibition independently. Thus, in our future studies, the present investigation will be followed up and complemented with mapping local circuit connections to single neurons located in specific V1 layers with LSPS and whole cell recordings.

It should be noted that during the experiments examining V1 response modulation by CPP, pharmacological application of glutamate receptor antagonists affected the effectiveness of laser photostimulation using caged glutamate. Furthermore, MNI-caged glutamate, like other caged compounds, at high concentrations (e.g., 2–3 mM used in two-photon uncaging experiments) can be an effective antagonist of GABAergic transmission (Fino et al., 2009). However, at the MNI-caged glutamate concentration used in the present and many previous studies (0.2 mM) (Shepherd et al., 2003, 2005; Wood et al., 2009; Shi et al., 2010), circuit inhibition appears to be preserved to a large degree in slices. In addition, the results obtained in photostimulation experiments were consistent with electrical stimulation experiments. Thus, we believe that the use of caged glutamate is not a major confound to the results presented with GABAa receptor blockers. An alternative to glutamate uncaging is photoactivation via channelrhodopsin-2 (ChR2) or other genetically encoded photosensitive molecules expressed in cortical neurons (Boyden et al., 2005; Kuhlman and Huang, 2008). Preliminary examinations using the latter optical approach indicate that population neuronal activity induced by ChR2 photoactivation have similar propagation patterns to that evoked by glutamate (Xu, 2011).

### V1 functional circuit connectivity

Using the functional mapping approach, the present study extended previous anatomical and physiological observations of V1 local circuits, and provided new information on mouse V1 circuit responses. We were able to examine the spatiotemporal profiles and topological arrangement of photostimulation-evoked VSD responses, and found that photostimulation-evoked VSD activation across different V1 locations was spatially discrete with specific laminar topography and largely constrained by vertical laminar connections. Although the VSD activity propagation had lateral horizontal spread to some degree, the vertical profiles of translaminar propagation were prominent. Our PCA examinations further indicated that the trajectories of the VSD responses from multiple laminar locations were grouped by their vertical columnar locations and arranged by their topography of stimulation sites. The physiological relevance of this organization, which is to maintain visual cortical topographical representations in radial “columns,” is elegantly demonstrated in this study.

Based on our mapping data, mouse V1 circuit organization is generally consistent with the proposed V1 laminar operational schemes for primates and cats (Gilbert, 1983; Callaway, 1998; Douglas and Martin, 2004). VSD responses initiated in layer 4 had strong projections to layers 2/3 and 5; the descending projection of layer 2/3 to layer 5 and the reciprocal ascending projection of layer 5 to layer 2/3 were clear. Nonetheless, compared to monkey V1 (Briggs, 2010), mouse V1 layers 4 had weak projections to layer 6, and layer 6 had sparse projections to upper layers. Perhaps this is not surprising as certain specific local circuit connections can be organized differently in different species. For example, similar to the present study, another LSPS mapping study reported the paucity of superficial layer input to layer 6 excitatory neurons in rat visual cortex (Zarrinpar and Callaway, 2006), in contrast to layer 6 neurons in primate visual cortex (Briggs and Callaway, 2001). The data acquired here was obtained from juvenile mice; although we anticipate that circuit connectivity patterns are conserved in mature cortex, future studies should address the issue of generalization.

The present study has derived detailed quantitative information for interlaminar functional connectivity. A simple neural model was constructed based on the quantification presented in Table 1. The model can simulate VSD data quantitatively in space and time, and allow for further analysis of V1 circuit responses. The modeling examination extends the experimental observation of interlaminar connections. In the control condition, the nearly absent layer 4 ↔ layer 6 connectivity reflects a few sparse connections. However, this cross-laminar activation can be enhanced with the removal of cortical inhibition in the model, approximating pharmacological conditions that permit trans-synaptic spread of activity biologically. As the layer 5 → layer 23 projection is present under control conditions but disappears when CPP is applied, we infer that, relative to other interlaminar projections, layer 5 → layer 23 projection is more plastic (hence its disappearance under CPP). In our model, given the interlaminar projection constraints of ‘columnar’ organization and data-informed overall strength, the qualitative distribution of that strength (the degree to which it is focused vs. broad) is determined by one parameter S, as described in the section “Materials and Methods.” Indeed, modeling these particular connection profiles with varying strength was necessary for the successful replication of the experimental results.

### Modulation of V1 circuit activity via GABA and glutamate receptors

Consistent with previous VSD imaging work in visual cortex (Tanifuji et al., 1994; Nelson and Katz, 1995; Yuste et al., 1997), we found that GABAergic inhibition was important in limiting spatiotemporal spread of VSD responses. Blocking GABAergic inhibition altered predominantly the late phase of population activity spread in mouse V1, as found in rat and mouse barrel cortex (Petersen and Sakmann, 2001; Laaris and Keller, 2002; Sato et al., 2008; Ikrar et al., 2012). However, bicuculline or gabazine strengthened excitation spread across all V1 laminar locations, particularly at layer 4, whereas blocking synaptic inhibition in the barrel cortex did not induce strong lateral spread between layer 4 barrel columns but in supragranular and infragranular laminar regions (Petersen and Sakmann, 2001; Laaris and Keller, 2002). This indicates that intracortical circuits in rodent V1 and primary somatosensory (S1) cortex are differentially organized with excitatory and inhibitory synaptic connections. Considering that rodent S1 cortex has a barrel columnar organization and rodent V1 lacks of anatomical and functional columnar organizations (e.g., orientation preference and ocular dominance columns) as described in highly visual mammals such as primates and cats (Ohki et al., 2005), the described columnar organization may be a contributing factor for differential intracortical organizations between rodent S1 and V1 cortex. Therefore, while the mouse V1 has been increasingly used to as a tractable system to address questions related to spatiotemporal visual processing (Niell and Stryker, 2008, 2010; Gao et al., 2010; Wang et al., 2011), it should be cautioned that spatiotemporal activity of population neuronal responses in local laminar circuits across species can differ due to the existence of anatomical or functional columnar organizations.

In this study, we further investigated how AMPA and NMDA receptor activation differentially contributed to neuronal population activity. Consistent with previous reports (Collingridge and Lester, 1989; Nishigori et al., 1990; Daw et al., 1993), we found that AMPA receptor activation was responsible for most significant neural transmission, as its antagonist CNQX alone essentially abolished photostimulation-evoked VSD responses and blocked translaminar propagation across V1 locations. In addition, the NMDA receptor antagonist, CPP preferentially suppressed photostimulation-evoked excitatory propagation at the peak phase, which fits with the slower kinetics but longer durations of NMDA receptor activation (Collingridge and Lester, 1989; Daw et al., 1993) and is consistent with the earlier report that NMDA receptors may differentially contribute to generation of neuronal action potentials or EPSPs with slow onsets in rat barrel cortex *in vivo* and in rat visual cortex *in vitro* (Nishigori et al., 1990; Armstrong-James et al., 1993). Therefore, NMDA receptor activation is important in modulating visual cortical circuit excitability in the mouse.

## Materials and methods

### Slice preparation

Thirty wild type C57/B6 mice were used for the experiments, in which one animal yielded one or two high-quality V1 slices with clear laminar and cytoarchitectonic features. All animals were handled and experiments were conducted in accordance with procedures approved by the Institutional Animal Care and Use Committee at the University of California, Irvine. To prepare living brain slices, animals (postnatal days 17–23) were deeply anesthetized with Nembutal (>100 mg/kg, i.p.), rapidly decapitated, and their brains removed. The occipital lobe was dissected, and visual cortical sections were cut in the coronal but slightly oblique (~15 degrees relative to the transverse) plane (which better preserves intracortical connections) at 400 μm with a vibratome (VT1200S; Leica Systems, Germany) in sucrose-containing artificial cerebrospinal fluid (ACSF) (in mM: 85 NaCl, 75 sucrose, 2.5 KCl, 25 glucose, 1.25 NaH2PO4, 4 MgCl2, 0.5 CaCl2, and 24 NaHCO3).

Slices were first incubated in sucrose-containing ACSF for 30 min to 1 h at 32°C, and upon the initial incubation period, transferred to recording ACSF (in mM: 126 NaCl, 2.5 KCl, 26 NaHCO3, 2 CaCl2, 2 MgCl2, 1.25 NaH2PO4, and 10 glucose) for the dye staining at room temperature. The slices were stained for approximately 1 h in a well oxygenated (95% O_2_–5% CO_2_) staining chamber containing ACSF with 0.02 mg/ml of the absorption VSD, NK3630 (Nippon Kankoh-Shikiso Kenkyusho Co., Ltd., Japan), then maintained in regular ACSF before use. The dye was chosen because of its good sensitivity, low bleaching and phototoxicity, as well as its preferential staining of neurons with a low affinity for glial cells (Konnerth et al., 1987; Jin et al., 2002). Neurons in the stained slices remained healthy after long sessions of VSD recordings as described previously (Xu et al., 2010). The NK3630 dye has been characterized in previous studies and has its peak signal-to-noise ratio centered around 705 nm (Jin et al., 2002).

### Photostimulation

Slices were visualized with an upright microscope (BW51X; Olympus, Tokyo, Japan) with infrared differential interference contrast optics. Electrophysiological recordings, photostimulation, and imaging of the slice preparations were done in a slice perfusion chamber mounted on a motorized stage of the microscope. At low magnification (4× objective lens; Olympus), brain slices were visualized under infrared bright-field transillumination; the slice images were acquired by a high resolution digital CCD camera (Q-imaging Inc, Austin, TX). Digitized images from the camera were used for guiding and registering photostimulation sites in cortical slices.

Stock solution of MNI-caged-l-glutamate (4-methoxy-7-nitroindolinyl-caged l-glutamate, Tocris Bioscience, Ellisville, MO) was added to 20–25 ml of circulating ACSF for a concentration of 0.2 mM caged glutamate. After 5–6 h of experimentation, the bath solution and MNI-glutamate was replaced. If needed, we corrected bath evaporation by adding a small amount of ACSF into the solution reservoir. To ensure a constant fluid level in the recording chamber of ~2.0 mm above the slice and to avoid small fluctuations in UV laser attenuation, the inflow reservoir was kept under a constant pressure of 3 PSI by using a pressure regulated perfusion system (Automate Scientific, Inc, CA).

For pharmacological experiments, to block GABAA receptors, 10–20 μM bicuculline methiodide or 20 μM gabazine (SR95531) (Tocris Bioscience, MO) was applied via bath solution. We excluded any slices exhibiting signs of spontaneous epileptic activity in the presence of GABAa receptor antagonists. Bath application of 10 μM CNQX (6-Cyano-7-nitroquinoxaline-2,3-dione disodium, Tocris Bioscience) and 10 μM CPP [3-(2-Carboxypiperazin-4-yl)-propyl-1-phosphonic acid, Tocris Bioscience] were used to block ionotropic glutamate receptors. With a perfusion flow rate of 1.5–2 ml/min, the drug application for 15 min was estimated to produce full effects, while the washout of 20 min was considered to remove the added drug from the recording solution. After washout, normal ACSF was restored with 0.2 mM caged glutamate.

Our overall system of combining LSPS with VSD imaging was described in our previous study (Xu et al., 2010). The spatial resolution of photostimulation was quantitatively estimated by using neuronal excitation profiles, which assess the spatial distribution of uncaging sites that produce action potentials in individually recorded neurons (Figure A1); similar approaches to assess the distribution of evoked neuronal excitability have been used by other groups (Shepherd et al., 2003; Shepherd and Svoboda, 2005; Weiler et al., 2008). Specifically, the spatial extent of effective photostimulation, R, was quantitatively estimated as the mean distance between the soma and the spike-generating sites weighted by the number of spikes per site [i.e., *R* = Σ (*r* × *n*)/Σn, where for each site, *r* is the distance to the soma and *n* is the number of spikes]. In addition, the photoexcitability of individual neurons was estimated by calculating the number of action potentials per cell per stimulus (S_AP_) over the area of π*R*^2^ centered on the somata. Under our photostimulation conditions (unless specified) (power level: 20 mW; pulse duration: 1–2 ms; caged glutamate concentration: 0.2 mM), the *R* for excitatory neurons across different V1 cortical layers did not differ significantly; its average value was 91.3 ± 1.2 μm within the window of analysis (75 ms post photostimulation) (*N* = 25 cells), and the average S_AP_ was 1.46 ± 0.02. Thus laser photostimulation in our experiments could provide spatially restricted neuronal activation, and offered a sufficient resolution for V1 laminar circuit mapping.

Based upon the excitation profile data, it was further estimated that each photostimulation activated approximately 250 neurons within ~90 μm of the laser uncaging center, as the number of spiking neurons is determined by the mouse cortical neuronal density, ρ, and the volume of excited neurons, V_exc_ [the product of photostimulation-evoked spiking area (π*R*^2^) and the most effective photostimulation penetration depth in the brain slices (75 μm on average for calculation)]. The neuronal density in mouse visual cortical layers 2/3, 4, 5, and 6 has been previously quantified, with the individual value being 14 × 10^4^, 19.52 × 10^4^, 8.32 × 10^4^, and 14.56 × 10^4^ neurons/mm^3^, respectively (Schuz and Palm, 1989). As the number of presynaptic spikes by each photostimulation could be reasonably estimated, the projection strength of these neurons can be quantitatively defined with the measurement of evoked activation by VSD imaging in their functionally connected regions (see below).

### Voltage-sensitive dye (VSD) imaging and data analysis

Upon triggering photostimulation, optical recording of VSD signals was performed by the MiCAM02 fast imaging system (SciMedia USA Ltd, Costa Mesa, CA) with a sampling rate of one frame per 2.2 ms (frame resolution 88(*w*) × 60(*h*) pixels). Under the 4× objective, the imaging field covered the area of 1.28 × 1.07 mm^2^ with a spatial resolution of 14.6 × 17.9 μm/pixel. The photostimulation interstimulus interval (ISI) was 8 s and the VSDI recording duration was 1000 frames (2.2 s) for each photostimulation trial. Optical signals evoked by photostimulation returned to baseline values within the allotted recording period. In addition, photostimulation evoked network activity showed good repeatability and low variations, judged by the consistency of maps across multiple repetitions. To supplement laser photostimulation, electrical stimulation through extracellular microelectrode or bipolar electrode stimulation (10–500 μA, 1 ms) in a specified cortical layer was used in some experiments.

VSD images were smoothed by convolving images with a Gaussian spatial filter (kernel size: 3 × 3 pixels; standard deviation (σ) size: 1 × 1 pixel) and a Gaussian temporal filter (kernel size: 3 frames; δ size: 1 frame). VSD signals were originally measured by the percent change in pixel light intensity [ΔI/I%; the % change in the intensity (ΔI) at each pixel relative to the initial intensity (I)]. In addition, signal amplitudes were expressed as standard deviations (SD) above the mean baseline signal for display and quantification. In the present study, single-trial voltage sensitive dye signals were of sufficiently high amplitudes and could be discerned from background noise. Data averaging of 2–3 trials was used for quantification unless specified. Images were displayed and initially analyzed using an acquisition and analysis software (BV-Analyzer; BrainVision, Tokyo, Japan). Further quantification and measurements were performed with custom-made Matlab Programs.

To perform quantitative analysis of evoked activation in image frames, the mean and standard deviation of the baseline activity of each pixel across the 50 frames preceding photostimulation was first calculated, and the activated pixel was empirically defined as the pixel with the amplitude ≥1 SD above the mean of the corresponding pixel's amplitude preceding the stimulation (equivalent to the detectable signal level in the original VSD maps of ΔI/I%). The activated pixels in response to photostimulation were measured across V1 layers. The temporal onset of VSD responses was determined by examining the time required for responses to reach above baseline at the uncaging epicenter. The defined phases of the optical response included the early phase (~11–33 ms after photostimulation), the peak phase (~55–77 ms after photostimulation), and the late phase (~99–121 ms after photostimulation).

Given the estimated number of presynaptic spikes generated by photostimulation in specified V1 layers (see above), the functional interlaminar projection strength (Q_proj_) can be estimated as Q_proj_ = *N*_postsynaptically activated neurons_/*N*_presynaptic AP_, where *N*_postsynaptically activated neurons_ is the measured number of activated pixel in their output laminar regions at the peak phase. As each pixel has a size of 14.6 μm × 17.9 μm, each activated pixel in the two-dimensional VSD image could account for ~2.7 neurons with the known neuronal density across mouse visual cortical layers and with an assumption that postsynaptic neurons are distributed in a volume with the axial depth comparable to presynaptic photostimulation penetration depth in the brain slices.

For statistical comparisons across >2 groups, we used the Kruskal-Wallis test (nonparametric one-way ANOVA) and the Mann-Whitney U test for group comparisons. Alpha levels of *P* ≤ 0.05 were considered significant. All the values were presented as mean ± SE.

### Principal component analysis of VSD image data

Before PCA was applied, the VSD image data had been processed as follows. Approximately 250 frames of raw VSD signals before the photostimulation onset were designated as a background response. A single background frame (60 × 88 pixels) was obtained by calculating the median over the background frames. The background frame was then subtracted from the evoked response VSD frames, designated as frames following the disappearance of the laser photostimulation. The evoked response frames were then filtered pixel-by-pixel temporally (acausal (two-sided) Gaussian filter; σ: one frame, filter support: 4 frames), and spatially (Gaussian filter; σ: 2 × 2 pixels, filter support 8 × 8 pixels). This choice of filter parameters represents a compromise between noise removal and spatio-temporal smearing of VSD evoked responses. All PCA and related image analyses were performed with custom-written Matlab scripts.

PCA was applied to a collection of *N* frames of VSD data, {*F*_1_, *F*_2_, …, *F*_*N*_}, where each frame is represented by an *m* × *n* matrix of VSD values (in this study *m* = 60, *n* = 88). The above collection may contain frames from a single or multiple concatenated VSD image datasets [the latter allows us to project individual data sets onto a common (global) basis and compare their projections (expansion coefficients) in a common phase space]. In the first step, the frames are reshaped into a vector form, *f*_*i*_ ∊ ℜ^5280×1^ (*i* = 1, 2,…, *N*), by stacking up vertically the columns of frame matrices. The covariance matrix, Σ, is then defined by: $\Sigma \propto \sum_{i=1}^{N}{\bar{f}}_{i}{\bar{f}}_{i}^{T}$, where ${\bar{f}}_{i}=f_{i}-\mu$, and $\mu =\frac{1}{N}\sum_{i=1}^{N}f_{i}$ is the mean of the collection. Note that the normalization of Σ by $\frac{1}{N}$ or $\frac{1}{N-1}$ is not necessary, as this merely scales the eigenvalues of Σ by a constant while not affecting its eigenvectors. The principal components are then taken as the eigenvectors of Σ corresponding to its largest non-zero eigenvalues. Since ∑ is a symmetric matrix (Σ = Σ^*T*^), its principal components are orthogonal and can be taken as basis vectors. If the frames {*F*_1_, *F*_2_, …, *F*_*N*_}, belong to multiple datasets, as is the case in the present study, this basis is referred to as the global basis (Senseman and Robbins, 1999, 2002; Nenadic et al., 2002, 2003). The presence of substantial correlation in the data will result in a small number of principal components {*v*_1_, *v*_2_, …, *v*_*M*_}, capturing a significant fraction of the data variance (in the present study *M* ≤ 5). In particular, it can be shown that this is the optimal *M*-dimensional subspace (of the original data space) in terms of captured variance (Jolliffe, 2002). Each frame vector, ${\bar{f}}_{i}$, is then represented in the new basis by a low dimensional vector α_*i*_ ∊ ℜ^*M* × 1^, defined by $\alpha_{i}=V^{T}{\bar{f}}_{i}$, where *V* is a matrix whose columns are the *M* principal components defined above.

Since Σ is the spatial covariance matrix, the principal eigenvectors {*v*_1_, *v*_2_, …, *v*_*M*_} represent the so-called spatial modes (i.e., basis images) of the data, whereas the expansion coefficients, α_*i*_, describe the temporal evolution of the data. Two, or more experimental data sets can then be compared by representing them in the common basis {*v*_1_, *v*_2_, …, *v*_*M*_}. In particular, their differences in terms of temporal evolution and the velocity of signal propagation can then be quantified and analyzed. Finally, an *M*th order approximation of the frame vectors can be obtained by the following reconstruction formula: ${\hat{f}}_{i}=\mu +V\alpha_{i}\;\left(i=1,2\ldots ,N\right)$. Likewise, an *M*th order approximation $\left\{{\hat{F}}_{1},{\hat{F}}_{2},\ldots ,{\hat{F}}_{N}\right\}$ of the original frames can be obtained by re-shaping the frame vectors ${\hat{f}}_{i}$ into their original matrix form.

### Computational modeling

We chose to model neuron population responses so that the model could be directly informed by the population-scale quantitative information provided by our VSD imaging experiments. For similar population-level neural modeling approaches, including one informed by VSD imaging data (Markounikau et al., 2010), the reader is referred to (Knight et al., 1996; Richardson et al., 2005; Zavaglia et al., 2006). In our model, at every time step, each compartment's excitation is updated as described in Equation (1), where *A*^(*t*)^_*i*,*j*_ is the activity level in the *j*th compartment of the *i*th row at time *t*, and *Z*^(*t*)^_*m*,*n*_ is the strength of excitatory signaling to other compartments by the *n*th compartment of the *m*th row at time *t*. The decay constant *C*_1_ (*C*_1_ = *C*_ReP_ + *C*_AutoI_) consists of the repolarization rate and auto-inhibition level, which together determine the rate at which the activation level of a compartment decays; removal of cortical inhibition in the presence of GABAa receptor antagonists, is implemented as the removal of *C*_AutoI_. The parameter *C*_2_ modulates the strength of intercompartmental signaling. The *e*^−|*n*−*j*| * *S*_*L*(*m*),*L*(*i*)_/*W*_*L*(*m*),*L*(*i*)_^ term decreases as a function of columnar distance between compartments, which reflects that signaling or connection strength between groups of neurons generally weakens with distance. The connection strength noise term, *R*^*i*,*j*^_*m*,*n*_, is a multiplier drawn from a triangular distribution spanning from 0 to 2 and centered about 1. The voltage dependence of the NMDA current is captured by *N*^(*t*)^_*i*,*j*_, which scales the input to a compartment according to whether that compartment is sufficiently activated for its voltage-gated NMDA channels to be open, therefore contributing to its further excitation. In Equations (1) and (4), the expression *L*(*x*) denotes the layer membership of row *x*; for example, *L*(2) = *L*23.

Our quantifications of activity spread (Table 1) indicate that for every sender-receiver pair of layers, there is a specific magnitude of excitatory signaling. For each layer pair, the activated pixel numbers in Table 1A are converted to the values in *W*(“weight”) according to $W_{L(m),L(i)}=\frac{\mu +SE}{Q}$, where μ and *SE* are the mean and standard error, respectively, of the strength (in pixels) of layer *m*'s activation of layer *i*, and *Q* is a conversion factor representing the ratio of the total number of pixels in VSD images to the number of compartments in the model. It was found empirically that increasing *W* from μ/*Q* to the value given above, together with the noise term *R*^*i*,*j*^_*m*,*n*_, enabled the replication of experimentally observed patchy off-column activation (Figure 1B). Meanwhile, the projections may vary with respect to whether their weight is spatially focused, or somewhat more broad and diffuse. This qualitative feature is captured by the values in matrix *S*, which equal 1 unless experimental results suggest otherwise, and for which lower values correspond to a broader and shallower projection that ultimately preserves total interlaminar projection weight (note that $W_{L\left(m\right),L\left(i\right)}=\int_{0}^{\infty }e^{\frac{-{\left|n-j\right|}^{*}S_{L\left(m\right),\;L\left(i\right)}}{W_{L\left(m\right),\;L\left(i\right)}}}\;*\;S_{L\left(m\right),L\left(i\right)}d\left|n-j\right|$. For example, setting *S*_*L*5,*L*23_ < *S*_*L*23,*L*5_ = 1 is consistent with our experimental findings of L23→L5 connectivity being denser than the reverse, and *in silico* is shown to account for the disappearance of L5→L23 activation in the absence of NMDA current enhancement (Figure 9E).

At every *t*, for all *i*:1–20 (loop through rows) & *j*:1–17 (loop through columns)

(1) $$\frac{dA_{i,j}^{\left(t\right)}}{dt}=-C_{1}A_{i,j}^{\left(t\right)}+LasE+C_{2}*N_{i,j}^{\left(t\right)}*\sum_{m=1}^{Rows}\sum_{n=1}^{Columns}e^{-\frac{\left|n\;-j\right|*S_{L\left(m\right),L\left(i\right)}}{W_{L\left(m\right),L\left(i\right)}}}*S_{L\left(m\right),L\left(i\right)}*Z_{m,n}^{\left(t\right)}*R_{m,n}^{i,j}$$

(2) $$Z_{i,j}^{\left(t\right)}=\frac{{\left(A_{i,j}^{\left(t\right)}\right)}^{2}}{{\left(A_{i,j}^{\left(t\right)}\right)}^{2}+C_{3}}$$

(3) $$LasE=\{\begin{array}{c} I*e^{-t/\tau }\text{ if }\left(i,j\right)=\left(g,h\right)\\ \text{0 otherwise} \end{array}$$

(4a) $$N_{i,j}^{\left(t\right)}=\{\begin{array}{c} \text{1 if }A_{i,j}^{\left(t\right)}>0.75^{*}T\;\\ n_{L(i)}\text{ otherwise} \end{array}$$

(4b) $$N_{i,j}=n_{L(i)}$$

The sigmoid form of Equation (2) is adopted as the relationship between a neuron population's activation level and its signaling to other populations is nonlinear, particularly when activation is very weak (signaling is approximately zero for a range of “weak” activations) or strong (at which point signaling saturates). Non-linearities similar to the sigmoid (e.g., Heaviside) have been used in other studies to relate population activation to population signaling (Knight et al., 1996; Richardson et al., 2005; Markounikau et al., 2010).

Laser stimulation is “aimed” at a single compartment with coordinates (*g*, *h*). In Equation (3), *LasE* represents the strength of the laser stimulus to a compartment's excitatory neurons, and *I* is the initial input strength due to the laser-uncaged glutamate. *LasE* is modeled as a decaying exponential approximating laser photostimulation. Equation (4) describes the scaling of input to a compartment according to whether or not the NMDA current is activated. Under normal conditions (4a), this activation is voltage-dependent; in simulations of CPP application, there is no NMDA current (4b). Because blocking NMDA conductance affects whether cells become sufficiently activated to be visualized, we reason that the NMDA current's voltage threshold is slightly lower than the dye image detection threshold (*T*, discussed further below). *n*_*L*(*i*)_ < 1 is a layer-specific constant, with *n*_*L*5_'s lower value reflecting a greater sensitivity to NMDA current block.

The system (1), (2), (3), and (4) was solved by discretization with *dt* = 0.15 (a simulation timestep putatively corresponds to 1 ms of a slice's response), *I* = 15, τ = 15, and *C*_1,2,3_ = 0.1, 0.5, 5000, and 0.6, respectively (when simulating bicuculine-treated slices, *C*_1_ = 0.03). While *n*_*L*23_ = *n*_*L*4_ = *n*_*L*6_ = 0.53, *n*_*L*5_ = 0.43, reflecting varying laminar concentration of NMDARs (Aoki et al., 1994). Stimuli were applied to the central column (*h* = 9) and central row of each layer (i.e., *g* = 3, 6, 11, and 17 for layers L23, L4, L5, and L6, respectively).

Last, compartmental activation levels are converted to simulated dye images as described by Equation (5), where *B*^(*t*)^_*i*,*j*_ represents the strength of VSD signal (color coded), *T* is the threshold for signal detection, and 5*T* is the threshold for signal saturation. The function chosen in Equation (5) is phenomenologically reflective of VSD's voltage dependence. The threshold *T* is set at one standard deviation above the average compartmental activation level across the pooled peak phase frames from stimulation of L23, L4, L5, and L6. After signal strengths are determined, we assigned to each compartment a 3 × 3 pixel square, and smoothed the signal (by a Gaussian filter; σ: 1 × 1 pixels, filter support 5 × 5 pixels) to reduce the salience of edges in our simulation output—sharp edges would be an artifact of our compartmental simulation method.

(5) $$B_{i,j}^{\left(t\right)}=\{\begin{array}{c} 0\text{ if }A_{i,j}^{\left(t\right)}<T\\ 5T\text{ if }A_{i,j}^{\left(t\right)}>5T\\ A_{i,j}^{\left(t\right)}\text{ otherwise} \end{array}$$

All model-related work was performed using Matlab (Mathworks; Natick, MA).

## Author contributions

Xiangmin Xu conceived the work, collected and analyzed data, and wrote the manuscript. Nicholas D. Olivas collected and analyzed imaging data, and helped prepare the manuscript. Zoran Nenadic developed the computer code for principal component analysis. Victor Quintanar-Zilinskas and Zoran Nenadic conducted modeling analysis and helped prepare the manuscript.

### Conflict of interest statement

The authors declare that the research was conducted in the absence of any commercial or financial relationships that could be construed as a potential conflict of interest.
